# Exploring Exact Effects of Various Factors on Chloride Diffusion in Cracked Concrete: ABAQUS-Based Mesoscale Simulations

**DOI:** 10.3390/ma16072830

**Published:** 2023-04-02

**Authors:** Ruiqi Zhao, Mengli Wang, Xuemao Guan

**Affiliations:** School of Materials Science and Engineering, Henan Polytechnic University, Jiaozuo 454003, China; wml2630019983@163.com

**Keywords:** chloride diffusion, concrete, damage zone, numerical simulation, ABAQUS

## Abstract

Chloride ion attack is a major cause of concrete durability problems, and existing studies have rarely addressed the effects of damage zones. In this paper, an improved mesoscale model including five phases was constructed using the finite element software ABAQUS to study the diffusivity of chloride ions in cracked concrete. It was found that the damage zone is negligible when the crack width is less than 50 μm, while the width and depth of the damage zone are about 15 times the crack width and 15% of the crack depth when the crack is greater than 50 μm. The results show that the diffusion of chloride is greatly influenced by the crack width, while it is little-influenced by the crack shape. Low water–cement ratio and adequate hydration of the concrete are also key factors affecting chloride diffusion. In contrast, regular rounded aggregates have a positive effect on reducing chloride diffusion compared to irregularly shaped aggregates, and this effect becomes weaker with increasing service time. In addition, the protective layer can effectively prevent the diffusion of chloride in concrete. Therefore, when designing marine concrete, efforts should be made to ensure that the concrete has a low water–cement ratio, adequate hydration, less cracking and a protective layer.

## 1. Introduction

Chloride-induced corrosion of steel bars is a major cause of reduced durability of marine reinforced concrete (RC) [1], and billions of dollars are spent annually to repair damage [2]. The accumulation of chloride ions on the steel surface is a prerequisite for corrosion [3]. Al-Sodani and colleagues constructed mathematical models and evaluated the long-term coefficients of chloride diffusion and apparent chloride diffusion [4,5]. To improve the performance of RCs, it is essential to study chloride diffusion behaviors and the exact influence of various factors behind them.

RCs have extremely complex structures. At the microscopic scale, RCs can be seen as a unity of a solid matrix and micropores. Chloride diffusion is strongly dependent on the microstructural features. Tan et al. [6] reported that pore volume accounts for 5.2–27.2% of concrete. Pivonka et al. used scale-transition analysis and predicted that chloride diffusivity in the pore solution is much smaller compared to bulk salt solutions due to the structuring of water molecules along the charged micropore surface [7]. These micropores can become larger and form interconnected structures, which evolve into cracks under external loads and thermal cycling during service [8].

Unlike microscopic models, concrete is usually modeled at the mesoscale as a unity of four phases, including artificial cracks (ACs), cement paste (CP), aggregates (AGGs) and interfacial transition zones (ITZ) [9]. It has been reported that the pores and cracks are the main channels through which chloride ions and seawater enter concrete [10], so they should have a great effect on chloride diffusion. Zhang et al. [11] reported that the durability of sound concrete was 2.5 times that of cracked concrete. Jung et al. [12] reported that the chloride diffusion coefficient increased with crack width. Naotunna et al. [13] reported that the width and depth of ACs were positively correlated to some extent. Furthermore, cracks may appear in various shapes. Li et al. [14] studied the chloride diffusion behavior through experiments and numerical simulations. However, the agreement between their experimental results and simulation results was not very good, especially at the deepest depth that chloride ions can reach. Peng and co-workers reported that the region around the AC had greater porosity than the bulk CP [15]. This region is called the damage zone (DZ). How does the DZ affect chloride diffusion in cement? The volume of CP accounts for 50–60% of RC [16]. The water–cement ratio and the degree of hydration of CP can affect the porosity, which in turn affects chloride diffusion in concrete [17]. How do they affect chloride diffusion? AGG can be considered impermeable due to its much smaller porosity than CP [18]. By treating aggregates as spheres, Wang et al. [19] reported that different volumes of aggregate could prevent chloride diffusion at different levels. In practical situations, aggregates have various shapes and sizes, which may affect the diffusion of chlorides by changing their diffusion paths or tortuosity [20]. In addition, RC is usually coated with a protective layer (PL), such as epoxy resin, to improve the durability of marine concrete [21]. Li et al. reported larger deviations between experimental and simulated results, and the former was larger than the latter in the initial simulation [14]. Therefore, protective layers will also be the focus of this paper. The answers to these fundamental questions are important for accurately understanding the diffusion behavior of chloride ions in concrete.

It is important to note that existing studies rarely consider the effect of damage zones. It is crucial to construct a suitable model to predict the exact effect of different factors on chloride diffusion in cracked concrete. In this work, an improved five-phase mesoscale model including DZs is proposed to investigate the exact effect of different factors on chloride diffusion in concrete. The paper will be organized as follows. First, the propagation of the DZ is determined based on the comparison of experimental and simulation results. The reliability of the model is further verified by another set of experimental results. Then, using this model, the effects of the width as well as shape of the ACs, the water–cement ratio, the degree of hydration, the shape of the AGG and the protective layer on chloride diffusion are systematically investigated. Finally, based on the in-depth understanding of the above fundamental issues, valuable information for designing marine concrete will be presented. These insights on the exact effect of different factors on chloride diffusion should provide useful information for the design of marine concrete with appropriate compositions.

## 2. Models and Numerical Simulations

### 2.1. Models and Simulations

#### 2.1.1. Construction of Models and Simulations

ACs were created by inserting copper sheets of different widths into cylindrical concrete specimens (diameter = 100 mm; height = 100 mm) in experiments [14]. The depth of the AC was constant (20 mm) and the width was variable, including 50 μm, 80 μm, 100 μm and 200 μm, respectively. The concrete was placed in a solution containing 1% chloride ions. The chloride migration coefficient was measured using a rapid chloride migration method [22].

**Figure 1:**
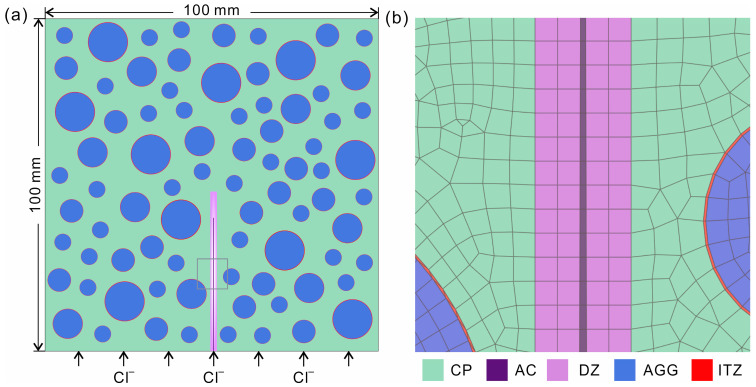
(**a**) Schematic diagram and (**b**) enlarged zone of the mesoscale model of concrete. Concrete consists of cement paste (CP), artificial cracks (ACs), a damage zone (DZ), aggregate (AGG) and an interface transition zone (ITZ). The mesh is a four-node linear heat transfer quadrilateral element sampled using the finite difference method.

The model was constructed as follows. First, a three-phase model containing AGG, ITZ and CP was constructed by running a homemade program in ABAQUS software. (version number: ABAQUS 2020) Then, the other two phases, DZ and AC, were created by splitting two isolated regions in the CP. The cross section of this model is shown in Figure 1. According to experimental results [14], the volume of the AGG was set to 43.8% of the model. The AGG is represented by circles with diameters of 5 mm, 7 mm, 9 mm and 12.5 mm, accounting for 24.2%, 24.6%, 23.2% and 28% of the AGG. The random distribution of the AGG is created by the Monte Carlo principle. The outermost region around the AGG is the ITZ. The thickness of the ITZ usually falls in the 20–50 μm range [23]. Here, the thickness of the ITZ is set to a constant of 50 μm in order to reduce the computation time. CP is mainly composed of cement, fine aggregate and water and is treated as a homogeneous phase. Its porosity and pore structure can be effectively adjusted by changing the water–cement ratio (*W*/*C*) [24]. In the real case, there is a DZ around the AC with a higher porosity than the CP [15]. Here, the DZ is treated as a homogeneous zone. The propagation of the DZ is closely related to the AC [25]. The width of the DZ is set to be 0, 5, 10, 15 and 20 times the width of the AC, and the depth of the DZ is 0, 5, 10, 15 and 20% deeper than that of the AC. Four cracks with widths of 50 μm, 80 μm, 100 μm and 200 μm, respectively, were constructed. The width and depth of the AC are denoted by *w*_AC_ and *D*_AC_, respectively.

In the numerical simulation, according to the mass diffusion equation and Fick’s law of diffusion, the concrete is considered to be fully saturated and only diffusion between seawater and concrete occurs [26]. After the model is assembled, the detailed simulation parameters are input in the analysis step. The transient analysis step is described by mass diffusion. Only the diffusion of chloride is allowed to take place on the bottom surface in the process of selecting boundary conditions (see Figure 1a). The mass diffusion module is integrated in the ABAQUS software [27]. The sample mesh is divided by the finite difference method [28] and the mesh type is a four-node linear heat transfer quadrilateral element with a size of 0.5 mm. All simulations are performed using the ABAQUS software.

#### 2.1.2. Detailed Information of Key Constituent Elements

Considering the differences in the five phases and their distinct effects on chloride diffusion, the following three factors are considered in constructing models:

(a) ACs

Under external loads, the width and depth of ACs are positively correlated within a certain range [29]. The width of the AC affects the openness of chloride ions into the concrete, which in turn affects the rate and quantity of chloride ions. To understand the effect of width, four ACs with widths of 49 μm, 102.9 μm, 210.7 μm and 392 μm were constructed, respectively. Six different shapes were considered in this work, including rectangle, ladder-shape, triangle, zigzag, Y-shape and branch. The areas of differently shaped ACs are the same.

(b) Water–cement ratio

The porosity and pore structure of concrete are closely related to the water–cement ratio (*W*/*C*) and the degree of hydration (*α*) [30]. It has been reported that the degree of hydration can reach as high as 0.89 when the *W*/*C* is 0.3 [31]. The increase in porosity leads to a decrease in concrete compactness, making it easier for chloride ions to penetrate into the concrete. To study the effect of *W*/*C* and *α* on chloride diffusion, four sets of samples with *W*/*C* of 0.3, 0.4, 0.5 and 0.6, respectively, were considered. For each sample, α was set to 0.8, 0.85 and 0.9, respectively.

(c) AGG shape

In real cases, AGG particles are not uniform in size and shape. To evaluate the possible influence of shape, AGGs with random shapes were also considered. The same gradation and volume ratio were adopted as that of the circular AGGs.

### 2.2. Diffusion Coefficients of Chloride Ions in Different Phases

The specific model in this work consists of five phases. The diffusion behavior of chloride ions in each phase is completely different. For CP, Zheng and colleagues obtained an analytical solution for the chloride diffusion coefficient (*D*_CP_, m^2^/s) based on the general effective medium theory, treating CP as a two-phase material composed of capillary pores and solid cement gel [32,33]:

*D*_CP_ = (2.14 × 10^−10^*V*_P_^2.75^)/[*V*_p_^1.75^(3 − *V*_P_) + 14.44 × (1 − *V*_P_)^2.75^] (1)

*V*_P_ = (*W/C* − 0.17*α*)/(*W/C* + 0.32) (2)

where *V*_P_ is the porosity of CP, *W*/*C* is the water–cement ratio and *α* is the degree of hydration.

For AC, many scientists have studied chloride diffusion behavior in cement cracks. Yoon [34] and co-workers reported that the chloride diffusion coefficient increased significantly with *w*_AC_ when the width was in the range of 30–200 μm, but when the width exceeds 200 μm, it was almost a constant as in free solution. The chloride diffusion coefficient in free solution is about 2.03 × 10^−9^ m^2^/s [35]. In this paper, based on experimental data available in refs. [36,37,38], a mathematical model is fitted as follows:

*D*_AC_ = (7.7 × 10^−6^*w*_AC_^3^ − 2.8 × 10^−3^*w*_AC_^2^
*+* 0.4*w*_AC_ − 7.4) × 10^−10^, 30 ≤ *w*_AC_ ≤ 200 µm (3)

For widths less than 30 µm or greater than 200 µm, the values of 30 µm and 200 µm were used, respectively.

For DZ, the chloride diffusion coefficient was set to be 20 times that of CP based on the experimental and simulation results reported by Peng and co-workers [15]. AGG was considered to be an impermeable entity with a diffusion coefficient of 0 [39]. ITZ was generally considered to be the separated phase, which contains higher porosity and lower cement than CP. Oh et al. [33] reported that the chloride diffusion coefficient of ITZ was about 4–8 times that of CP when the thickness of ITZ was 20 μm. Bourdette and colleagues concluded that the effective diffusion coefficient of ITZ was 6–12 times that of CP [40]. Xie et al. [41] reported that the coefficient was related to the thickness of ITZ, which is about 10 times that of CP when the thickness is 20 μm. Sun reported that the actual thickness of ITZ ranges from 30 μm to 80 μm [9]. In this work, the ITZ thickness was set to 50 μm, and the chloride diffusion coefficient of ITZ was 10 times that of the CP.

### 2.3. Determination of DZ

A key improvement of our model is to treat the DZ around the AC as part of the concrete. Therefore, we first need to determine the propagation of the DZ. Four ACs were constructed with the same width (50 μm, 80 μm, 100 μm and 200 μm) and depth (fixed at 20 mm) as reported in [14]. Peng et al. also considered the DZ and assumed that the width of the DZ is several times wider than the AC and the depth of the DZ is 10 mm deeper than the AC [15]. The propagation of the DZ is closely related to the width and depth of the AC. In this work, the width of DZ is set to be 0, 5, 10, 15 and 20 times the width of the AC, and the depth of DZ is 0, 5%, 10%, 15% and 20% deeper than that of AC, respectively. The propagation of DZ can be described simply by a pair of numbers (width, depth). Therefore, the resulting DZs are denoted as (0, 0), (5, 5), (10, 10), (15, 15) and (20, 20), respectively.

**Figure 2:**
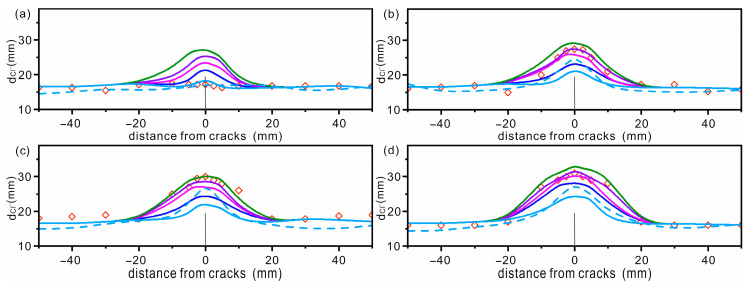
The diffusion depth (d_Cl_^−^) of chloride in concrete with AC widths of (**a**) 50 μm, (**b**) 80 μm, (**c**) 100 μm and (**d**) 200 μm, respectively. The center of the AC is set to 0. The DZ regions are described by a pair of numbers. Light blue, blue, pink, purple and green represent a diffusion of chloride in concrete with DZs of (0, 0), (5, 5), (10, 10), (15, 15) and (20, 20), respectively. The diamonds and dash lines represent the experimental and simulation results of [14] (reproduced with permission from [14], copyright 2016 Elsevier).

The concrete was epoxy-coated except for the bottom surface and placed in a 1% chloride solution to ensure that the chloride diffused only in the direction perpendicular to the bottom. The chloride diffusion coefficient in CP is 1.34 × 10^−11^ m^2^/s, and is 6.56 × 10^−10^ m^2^/s, 1.06 × 10^−9^ m^2^/s, 1.23 × 10^−9^ m^2^/s and 2.22 × 10^−9^ m^2^/s in each of the four ACs. The simulation time was 37 days. The obtained diffusion depths of chloride in concrete with 4 different ACs are shown in Figure 2. The simulation results and experimental results in [14] are also shown in Figure 2 to obtain an appropriate DZ. For the AC with a width of 50 μm, it can be seen that the simulated curve without a DZ agrees well with the experimental data, while the curves considering the DZ deviate from the experimental results, and the deviation becomes more serious with the increasing DZ (see Figure 2a). The good agreement between the simulation results without a DZ and the experimental results suggests that, for ACs smaller than 50 μm, DZ does not need to be considered. Wang et al. [42] and Aldea et al. [43] independently reported no difference in chloride diffusion between perfect concrete and concrete with AC widths less than 50 μm. For ACs with widths of 80 μm and 200 μm, the simulation curves with DZs are closer to the experimental data, and the data with a DZ of (15, 15) agree well with the experimental data (see the purple curve in Figure 2b,d). For ACs with a width of 100 μm, the simulation curve with a DZ of (20, 20) is closer to the experimental data, but the difference between the two curves with DZs of (15, 15) and (20, 20) is small (see Figure 2c). Therefore, in subsequent simulations, the DZ is not considered when the crack width is less than 50 μm, and the DZ propagation of (15, 15) is considered when the crack width is greater than 80 μm.

## 3. Results and Discussion

### 3.1. Reliability of Numerical Models

**Figure 3:**
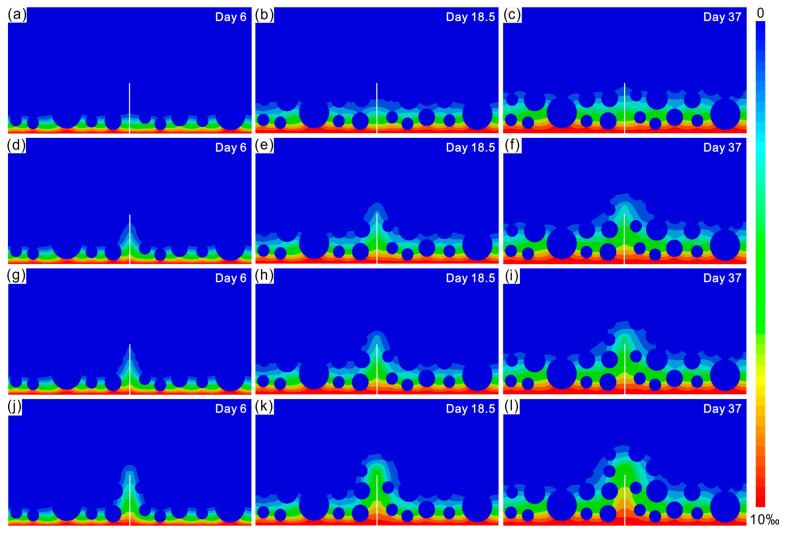
Distribution of chlorides in concrete with AC widths of (**a**–**c**) 50 μm, (**d**–**f**) 80 μm, (**g**–**i**) 100 μm and (**j**–**l**) 200 μm after immersion for different times. AC is highlighted with a white line. Colors from blue to red represent chloride ion concentrations from 0 to 10‰.

**Figure 4:**
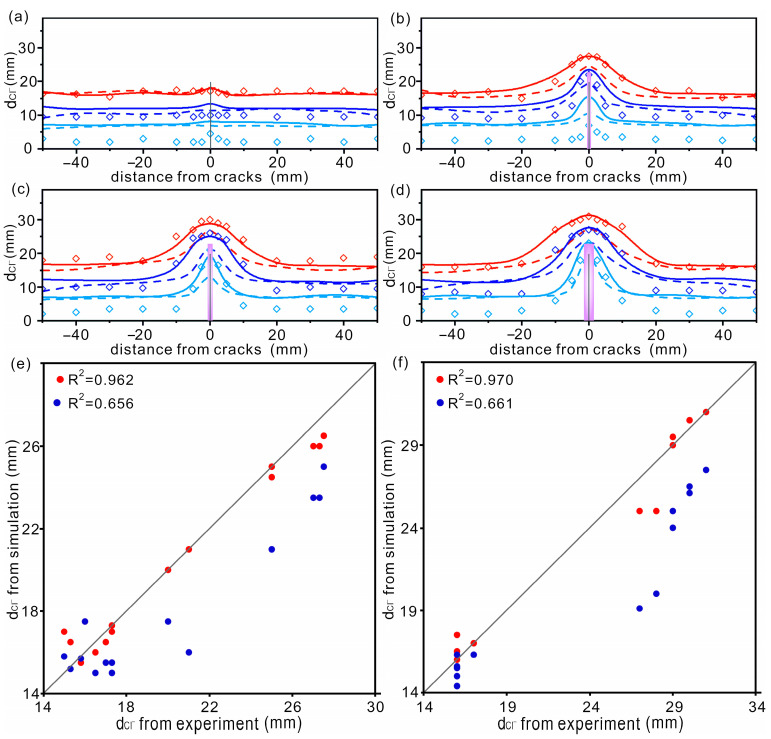
(**a**–**d**) The outermost diffusion contours of chloride in concrete after immersion for different times. The widths of ACs are (**a**) 50 μm, (**b**) 80 μm, (**c**) 100 μm and (**d**) 200 μm, respectively. Light blue, blue and red represent data collected at 6 d, 18.5 d and 37 d, respectively. The simulation results in this work are shown as solid lines. The simulation results and experimental results taken from [14] (reproduced with permission from [14], copyright 2016 Elsevier) are shown with dashed lines and diamonds, respectively. The DZ is not considered in case with AC width of 50 μm (**a**). The purple region in panels (**b**–**d**) represents the DZ described by (15, 15). (**e**,**f**) Correlation between simulated d_Cl_^−^ and measured chloride diffusion depth at 37 d in concrete with AC widths of 80 μm (**e**) and 200 μm (**f**). The red and blue dots represent simulated data in the present work and other works [14]. The experimental results were also taken from [14].

The resulting numerical model was used to simulate chloride diffusion in concrete. Data were collected on days 6, 12, 18.5, 25, 31 and 37, respectively. For clarity, the chloride distributions obtained at 6 days, 18.5 days and 37 days are shown in Figure 3. Colors from blue to red represent chloride concentrations from 0 to 10‰. The outermost diffusion contours at different times and correlation plots are shown in Figure 4. For comparison, the simulation results (dashed lines) and experimental results (diamonds) of [14] are also shown in Figure 4 to verify the reliability of the numerical model. In order to show the quantitative validation between the simulation results and experimental data, here we take the case of AC widths of 80 um and 200 um; the correlation between the present work and the experimental data [14] is shown in Figure 4e,f. The simulation results from [14] are also shown for comparison. It can be seen that this model can describe the diffusion behavior of chloride well. The following points can be seen from Figure 4:

(a) The simulation results of this work are closer to the experimental data than [14], especially in the region around the AC. Good agreement is more evident at all simulation times in cases with larger ACs (see the solid lines and the point data in Figure 4c,d).
(b) There is a difference between the simulation results and the experimental data at the initial simulation time, which is more pronounced in the case with a smaller AC (see Figure 4a). This difference becomes weak with increased soaking time. The reason for this difference will be discussed further in the section below.

The above points show that the model can accurately describe the chloride diffusion behavior in concrete, especially when the immersion time is long and the AC is large. In the next section, we will turn to study the influence of different factors on the diffusion of chloride ions.

### 3.2. The Effect of ACs

#### 3.2.1. Effect of ACs with Different Widths and Depths

**Figure 5:**
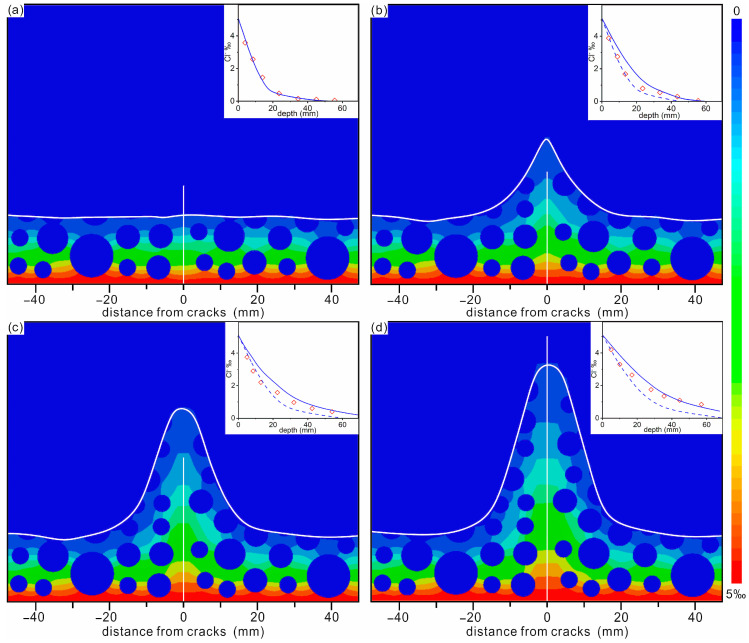
Diffusion cloud maps of chloride ions in concrete containing ACs with widths of (**a**) 49.0 µm, (**b**) 102.9 µm, (**c**) 210.7 µm and (**d**) 392 µm. The outermost diffusion contours are highlighted with white lines. The chloride ion concentrations along the direction highlighted by white arrows are shown as insets. The experimental data of [37] (reproduced with permission from [37], copyright 2007 Elsevier) and simulation results without DZs are also shown as diamonds and dashed lines in the insets for comparison. Colors from blue to red represent chloride ion concentrations from 0 to 5‰.

In general, the wider the AC, the deeper the AC. To study the effect of cracks of different widths and depths and to further validate the reliability of the model, we constructed another set of ACs reported in [37]. The width and depth of DZ are calculated from the above fitting parameters; for ACs with a width less than 50 μm, DZ is ignored, and for ACs with a width greater than 50 μm, the width and depth of DZ are 15 times and 15% of AC, respectively. The diffusion coefficient of chloride ions in CP is 2.34 × 10^−11^ m^2^/s. The diffusion coefficients of chloride in different ACs can be calculated from Equation (3). The details of the four AC, DZ and chloride diffusion coefficients are summarized in Table 1. The simulation time is 30 days, which is equivalent to 30 days in the experiment. The diffusion cloud maps of chloride ions in concrete are shown in Figure 5. The outermost diffusion contours are highlighted with white lines for clarity.

It clearly shows that the diffusion depth increases with the AC depth (Figure 5). Taking ACs with widths of 49.0 μm and 392 μm as examples, the diffusion depths in the center of the ACs are 20.2 mm and 67.4 mm, respectively. Another feature presented in Figure 5 is that the concentration of chloride ions decreases with diffusion depth. The concentration of chloride ions can be measured experimentally. To quantitatively present the concentration change, the concentrations along the AC direction are shown as insets in each panel (see the solid lines in the inset of each panel in Figure 5).

It can be observed that the chloride concentration drops sharply with increasing depth, and the rate of decline decreases with increasing depth. For the same diffusion depth, the concentration of chloride ions increases with AC width. Taking a depth of 10 mm as an example, the chloride ion concentrations in concrete with ACs of 49.0 μm and 392 μm are 2.13‰ and 3.82‰, respectively. Note that the chloride concentration in the free solution was set to 1%. Therefore, the width and depth of ACs have a significant effect on the chloride diffusion in concrete.

To further demonstrate the good reliability of our model, the experimental concentration of chloride ions [37] and the simulation results without DZ are also shown in the inset. It can be seen that the simulation results without DZ deviate from the experimental data as the depth increases.

The simulation results of our model can describe the diffusion behavior of chloride ions well, especially in deeper regions (see data represented by solid lines and diamonds in each inset). These results again demonstrate the good reliability of our model and the necessity of considering the DZ effect when simulating chloride diffusion in concrete, especially with larger ACs.

#### 3.2.2. Effect of the Shape of ACs

**Figure 6:**
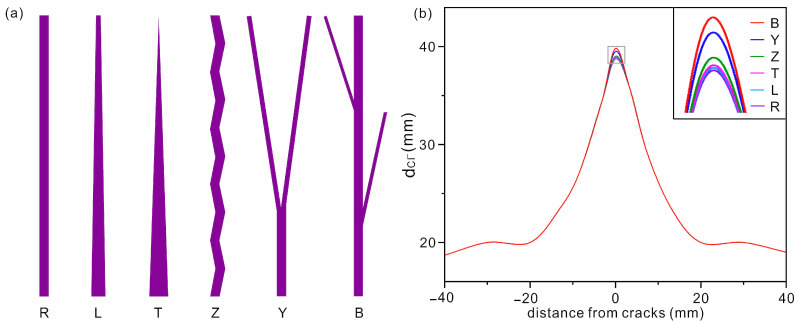
(**a**) Schematic diagram of AC with different shapes, including rectangular (R), ladder-shaped (L), triangular (T), zigzag (Z), Y-shaped (Y) and branched (B). The width and length of the rectangular ACs are 102.9 µm and 36.6 mm, respectively. The area of these six ACs is set to a constant 3.77 mm^2^. (**b**) Diffusion depth of chloride ions in concrete with different shapes of ACs. The enlarged zone around the ACs is shown as an inset.

In the above discussion, the shape of the AC was simplified to a rectangle to save computational cost. However, in practical situations, ACs have various shapes, which may in turn affect chloride diffusion in concrete. To study the effect of AC shape, we here constructed ACs of six shapes, including rectangular (R), ladder-shaped (L), triangular (T), zigzag (Z), Y-shaped (Y) and branched (B) (see Figure 6a for the schematic diagram of ACs). The rectangular AC is 102.9 µm × 36.6 mm. The area of these six ACs was set to a constant of 3.77 mm^2^.

The simulation time was 30 days. The outermost contours of the diffusion depth are shown in Figure 6b. For clarity, the enlarged zone around the ACs is shown as inset in Figure 6b. It can be seen that chloride ions have a deep diffusion depth in concrete with branched, Y-shaped and zigzag ACs in the order of AC(B) > AC(Y) > AC(Z). For concrete with the other three types of AC (AC(R), AC(L) and AC(T)), the diffusion depth is almost the same. The difference in diffusion depth between the maximum AC(B) and the minimum AC(R) is 1 mm, which corresponds to 2.5% of the diffusion depth. The perimeters of branched ACs and rectangular ACs are 122.2 mm and 73.4 mm, respectively.

The small difference may be attributed to the difference in the contact area between the solution and concrete around the ACs. Overall, the shape of ACs has little effect on the chloride diffusion in concrete. Therefore, it is reasonable to simplify ACs to a rectangle in the simulation.

### 3.3. Influence of Water–Cement Ratio and Degree of Hydration

The water–cement ratio (*W*/*C*) is an important parameter that determines the mechanical properties of concrete, such as strength, hardness and durability, and has a great influence on the permeability of concrete [44]. The degree of hydration (*α*) is another parameter closely related to porosity [45]. Therefore, it is very important to reveal the effects of water–cement ratio and degree of hydration on the chloride diffusion behavior, and on this basis, design concrete to reduce the permeability of chloride ions. In this paper, four commonly used *W*/*C* ratios and three α for each *W*/*C* ratio are considered. A rectangular AC with a width and length of 102.9 µm and 36.6 mm, respectively, was introduced into the concrete. The diffusion coefficient of chloride ions in AC is 12.5 × 10^−10^ m^2^/s. The diffusion coefficients of chloride ions in CP (*D*_CP_, m^2^/s) calculated from Equations (1) and (2) are summarized in Table 2. The simulation time was 90 days.

**Figure 7:**
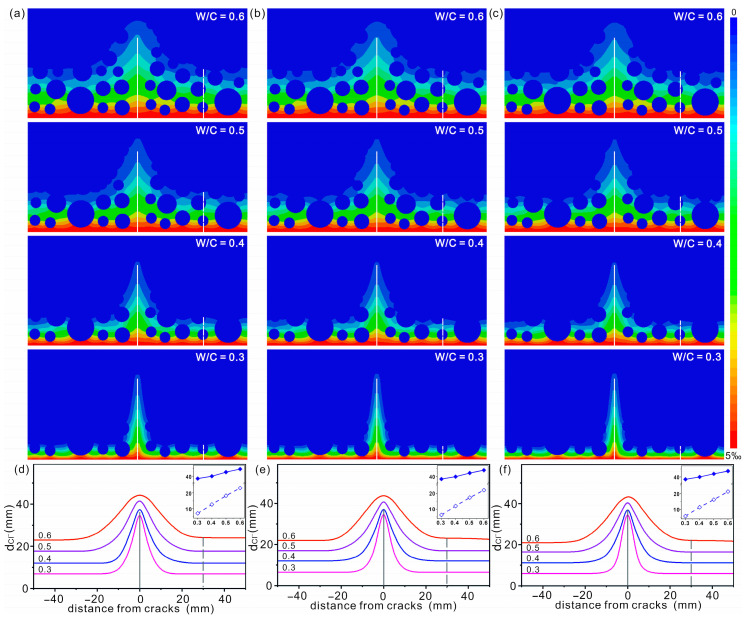
Diffusion cloud maps (**a**–**c**) and the outermost diffusion contours (**d**–**f**) (left to right) of chloride in concrete with hydration degrees of 0.8, 0.85 and 0.9, respectively. AC is shown as a solid white/black line. The other position where diffusion data was collected is highlighted with a dashed line. The chloride diffusion depths versus different *W*/*C* ratios with x = 0 mm (solid) and x = 30 mm (hollow) are shown as inset.

The chloride diffusion cloud maps in concrete with different *W*/*C* ratios and α are shown in Figure 7a–c. The water–cement ratio, degree of hydration, chloride diffusion coefficient in CP, diffusion depth along AC and the location 30 mm away from AC are summarized in Table 2.

A quick look at the diffusion maps shows that chloride diffusion in concrete increases with *W*/*C*. To show the effect of *W*/*C* more clearly, the outermost diffusion contours with the same α are presented in Figure 7d–f, respectively. It clearly shows that the chloride diffusion depth in concrete increases with an increasing *W*/*C* ratio, regardless of whether the region contains cracks or not. Taking *α* = 0.8 as an example, when W/C ratios are 0.3, 0.4, 0.5 and 0.6, the diffusion depths at x = 30 mm are 7 mm, 12.7 mm, 18.2 mm and 23.1 mm, respectively. The diffusion depth increases up to 16 mm. For concrete with the other two *α*, the increase is 15.5 mm (*α* = 0.85) and 15 mm (*α* = 0.9), respectively. To further clearly present the relationship between diffusion depth and water–cement ratio, the diffusion depths in concrete with different *W*/*C* ratios are shown as inset in Figure 7d–f. It clearly shows that the diffusion depth of chloride ions increases almost linearly with the *W*/*C* ratio.

**Figure 8:**
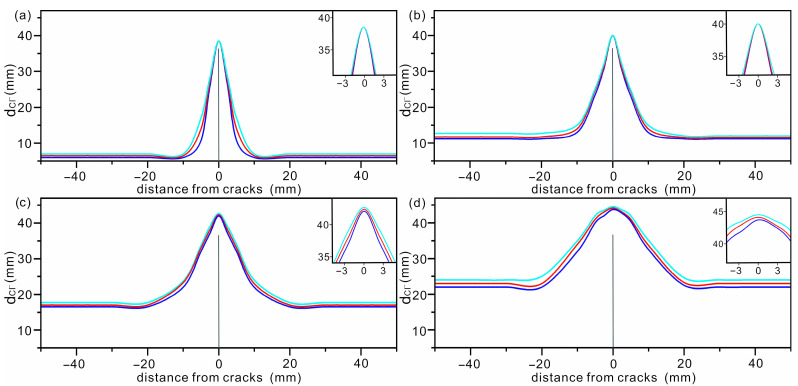
The outermost diffusion contours of chlorides in concrete with water–cement ratio of (**a**) 0.3, (**b**) 0.4, (**c**) 0.5 and (**d**) 0.6, respectively. AC is shown as a solid black line. Bright blue, red and blue represent data collected with degree of hydration of 0.8, 0.85 and 0.9, respectively.

Compared with *W*/*C* ratio, the degree of hydration has much less effect on chloride diffusion. To show this effect more clearly, the chloride diffusion depth in concrete with the same *W*/*C* ratio is shown in Figure 8. The enlargement of the deepest region around the AC is shown as an inset. It can be observed that for the concrete with the same *W*/*C* ratio, the diffusion width is slightly larger in case with smaller *α*, and this trend is more pronounced when the *W*/*C* ratio is high. Another feature observed in Figure 8 is that the diffusion depth is hardly affected by the degree of hydration. Greater width and depth mean higher permeability of chloride ions in concrete. Therefore, concrete designed for the marine environment should be that with a low water–cement ratio under the premise of ensuring sufficient hydration. Han also reported that *W*/*C* was a key parameter for designing concrete for marine environments [46].

### 3.4. Influence of Aggregate Shapes

In the above discussion, all the simulations were performed by simplifying the AGGs to a regular sphere; however, in the real cases, the AGGs are usually irregular polyhedrons with different sizes and shapes. To evaluate the effect of different shapes on chloride diffusion in concrete, we modified the model here to replace regular AGGs with randomly shaped AGGs.

**Figure 9:**
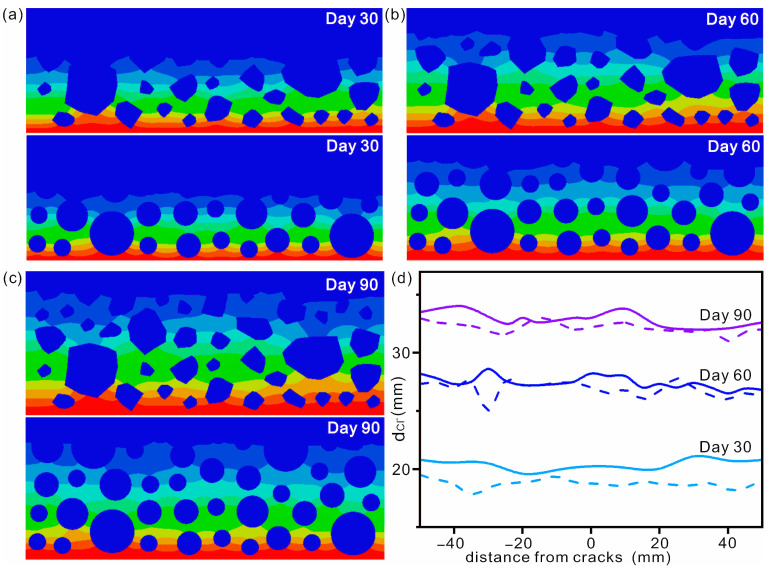
Diffusion cloud maps of chloride ions in concrete with random polyhedral AGGs and circular AGGs for (**a**) 30 days, (**b**) 60 days and (**c**) 90 days. (**d**) Outermost diffusion contours of chloride in concrete after immersion for different times. The solid and dashed lines represent the AGGs with random polygons and regular spheres, respectively.

The construction principle is to ensure the same gradation and volume ratio are the same as those of regular AGGs. Data were collected at 30, 60 and 90 days, respectively. The diffusion cloud maps of chloride are shown in Figure 9a–c, respectively. To show the difference in chloride diffusion more clearly, the outermost contours are shown in Figure 9d. It can be seen that the chloride diffusion depth in concrete with random-shaped AGGs is larger than that of circular AGGs. It should be noted that the volume ratio of the two types of AGGs is equal. Another feature presented in Figure 9d is that the diffusion difference becomes smaller with increased immersion time. For concrete in marine environments, it can last for decades. From a long-term perspective, such as the durability of the whole system, the AGG shape has a negligible effect on chloride diffusion. That is, when studying the corrosion of concrete by chloride ions, the AGGs can be simplified to simple spheres. Li and colleagues also reported the diffusion behavior of chloride in concrete by treating AGGs as spheres, and obtained results consistent with experiments [47]. However, corrosion is a cumulative process and the initial entry of chloride ions is a prerequisite for corrosion. From the point of improving the overall performance of concrete, regular-shaped AGGs should be better.

## 4. Further Discussion

An obvious difference between the simulation results and the experimental results is that, in the initial simulation stage, the simulation results are larger than the experimental results (see Figure 4). This difference suggests that chloride diffuses more slowly in the early stage.

**Figure 10:**
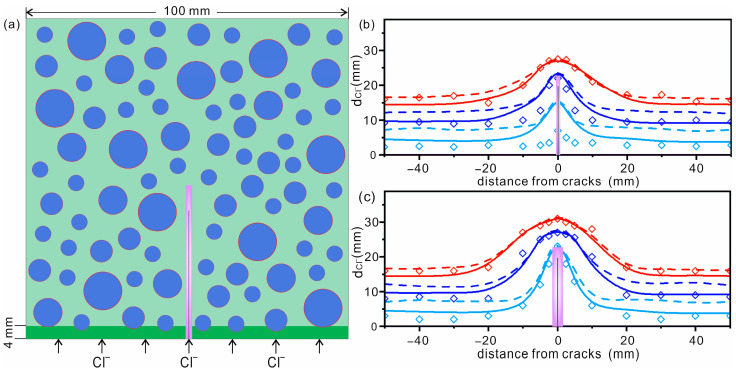
(**a**) The same mesoscale schematic model of concrete is considered, except for a 4 mm thick protecting layer (PL, highlighted with green zone). (**b**,**c**) Outermost diffusion contours of chloride in concrete with AC widths of 80 μm (**b**) and 200 μm (**c**). Light blue, blue and red represent data collected at 6, 18.5 and 37 days, respectively. Simulated data with and without the PL are shown as solid and dashed lines, respectively. Experimental results (diamonds) taken from [14] (reproduced with permission from [14], copyright 2016 Elsevier) are also shown for comparison. The solid black line and the purple zone represent the AC and DZ, respectively.

In real cases, the concrete used in marine environments is coated with protective layer to reduce seawater corrosion [48]. Schueremans et al. [49] reported that the thickness of protective layers in surface-treated concrete was 1–6 mm. The PL may be a factor hindering chloride diffusion in the early stages. To explore the reasons for the difference between the simulated and experimental results at the initial stage, a 4 mm thick PL was considered in the modified mesoscale model (see Figure 10a for details). Wang and co-workers reported that the chloride diffusion coefficient in PL is about 1/3 that of CP [50]. Here, the diffusion coefficient of chloride in PL is simplified to one third of that in CP. Other parameters remain unchanged. The ACs with widths of 80 μm and 200 μm were constructed. The simulation time was 37 days.

The obtained outermost contours of chloride diffusion with and without PL are shown in Figure 10b,c, respectively. Experimental data from [14] are also shown for comparison. It can be seen that the simulation results considering PL are more consistent with the experimental data in the initial stage (see Figure 10b,c), and the agreement is better in the case with a larger AC. It should be noted that the diffusion coefficient of chloride in the PL is roughly treated as one-third that of CP. The good agreement suggests that it is necessary to consider the effect of a PL in the simulations to better describe chloride diffusion behaviors. Of course, more simulation and experimental work will be needed in the near future to reveal the exact effects of PL.

## 5. Conclusions

In summary, an improved mesoscale model including a damage zone was proposed to simulate the diffusion behavior of chloride ions in concrete. The propagation of the DZ was determined from experimental data. Using this model, we studied the exact effects of cracks, water–cement ratio and aggregate shape on the chloride diffusion in concrete. The following results can be obtained:

(1) The damage zone around the crack is negligible when the crack width is less than 50 μm, but is not negligible when the crack width is greater than 50 μm. The propagation of the damage zone is about 15 times and 15% of the crack width and depth, respectively;
(2) The chloride diffusion in concrete is greatly affected by the depth and width of cracks, and is hardly affected by the shape of cracks. Both the diffusion depth and concentration of chlorides increase with crack width;
(3) The water–cement ratio also has a significant effect on the chloride diffusion in concrete. As the ratio increases, the diffusion depth increases almost linearly. For cement with the same water–cement ratio, the degree of hydration has little effect on the chloride diffusion depth, but can affect the diffusion width. The higher the degree of hydration, the narrower the diffusion width;
(4) The AGG shape has a small effect on chloride diffusion. The diffusion in concrete with irregular aggregate is a little more serious than that in concrete with regular aggregate. The difference becomes smaller as the soaking time increases.

## Figures and Tables

**Table 1:** The width and depth of ACs, DZs and the diffusion coefficient (*D*_AC_) of chloride ions in different ACs. The width and depth of ACs/DZs are denoted by *w*_AC_/*w*_DZ_ and *d*_AC_/*d*_DZ_, respectively.

* ACs	*w*_AC_ (µm)	*d*_AC_ (mm)	*w*_DZ_ (mm)	*d*_DZ_ (mm)	*D*_AC_ (×10^−10^, m^2^/s)
a	49.0	28.1	0	0	6.38
b	102.9	36.6	1.54	42.1	12.5
c	210.7	47.3	3.16	54.4	22.2
d	392	75.4	5.88	86.7	22.2

* The ACs were constructed with the same width and depth as reported in [37].

**Table 2:** Parameters including diffusion coefficient in CP (*D*_CP_, ×10^−12^, m^2^/s) and diffusion depth (*d*_Cl_^−^, mm) of chloride in concrete with different water–cement ratio (*W*/*C*) and degree of hydration (*α*).

***W*/*C***	0.3			0.4			0.5			0.6		
***α***	0.8	0.85	0.9	0.8	0.85	0.9	0.8	0.85	0.9	0.8	0.85	0.9
***Parameters***												
***D*** **_CP_**	0.86	0.71	0.58	2.98	2.61	2.28	6.59	5.95	5.37	11.46	10.57	9.73
* ***d*_Cl_^−^**	38.5	38.5	38.5	40	40	40	42.6	42.3	42	44.5	44.1	43.7
** ***d*_Cl_^−^**	7	6.3	5.7	12.7	12	11.4	18.2	17.3	16.2	23.1	22	21.3

* Data collected along the center of ACs; ** data collected at a location 30 mm from AC (highlighted with dashed lines in Figure 7).

## Data Availability

The data required to reproduce are available to obtain from the corresponding authors.

## Abbreviations

**Terms**	**Abbreviations**
reinforced concrete	RC
cement paste	CP
artificial cracks	AC
damage zone	DZ
aggregate	AGG
interface transition zone	ITZ
water–cement ratio	*W/C*
degree of hydration	*α*
diffusion coefficient	*D*
protective layer	PL
width of artificial cracks	*w*_AC_
depth	*d*
